# Mitochondria in the Nuclei of Rat Myocardial Cells

**DOI:** 10.3390/cells9030712

**Published:** 2020-03-14

**Authors:** Chupalav M. Eldarov, Irina M. Vangely, Valeriya B. Vays, Eugene V. Sheval, Susanne Holtze, Thomas B. Hildebrandt, Natalia G. Kolosova, Vasily A. Popkov, Egor Y. Plotnikov, Dmitry B. Zorov, Lora E. Bakeeva, Vladimir P. Skulachev

**Affiliations:** 1 A.N. Belozersky Institute of Physico-Chemical Biology, Lomonosov Moscow State University, 119991 Moscow, Russia; chupalav@protonmail.ch (C.M.E.); sim870@mail.ru (I.M.V.); valeriya.vays@yandex.ru (V.B.V.); sheval_e@belozersky.msu.su (E.V.S.); popkov.vas@gmail.com (V.A.P.); plotnikov@belozersky.msu.ru (E.Y.P.); zorov@belozersky.msu.ru (D.B.Z.); bakeeva@belozersky.msu.ru (L.E.B.); 2 Department of Reproduction Management, Leibniz-Institute for Zoo and Wildlife Research, Alfred-Kowalke-Str. 17, 10315 Berlin, Germany; holtze@izw-berlin.de (S.H.); hildebrandt@izw-berlin.de (T.B.H.); 3 Institute of Cytology and Genetics, Siberian Branch of Russian Academy of Sciences, Novosibirsk 630090, Russia; kolosova@bionet.nsc.ru; 4 Faculty of Bioengineering and Bioinformatics, Lomonosov Moscow State University, 119992 Moscow, Russia

**Keywords:** intranuclear mitochondria, healthy cells, electron and confocal microscopy, heart

## Abstract

Electron microscopic study of cardiomyocytes taken from healthy Wistar and OXYS rats and naked mole rats (*Heterocephalus glaber*) revealed mitochondria in nuclei that lacked part of the nuclear envelope. The direct interaction of mitochondria with nucleoplasm is shown. The statistical analysis of the occurrence of mitochondria in cardiomyocyte nuclei showed that the percentage of nuclei with mitochondria was roughly around 1%, and did not show age and species dependency. Confocal microscopy of normal rat cardiac myocytes revealed a branched mitochondrial network in the vicinity of nuclei with an organization different than that of interfibrillar mitochondria. This mitochondrial network was energetically functional because it carried the membrane potential that responded by oscillatory mode after photodynamic challenge. We suggest that the presence of functional mitochondria in the nucleus is not only a consequence of certain pathologies but rather represents a normal biological phenomenon involved in mitochondrial/nuclear interactions.

## 1. Introduction

In 1958, Australian electron microscopists H. Hoffman and G. W. Grigg, when analyzing ultrathin sections of lymph nodes of adult mice found clustering of mitochondria around the concavities in the nuclear membrane, some lying in very close juxtaposition to the membrane [1]. They even suggested the presence of mitochondria inside of the nucleus but given that the quality of electron microscopic images was not perfect, this suggestion stayed hypothetical. However, in 1960, H. Mori described mitochondria in nuclei of cells from four types of ascites cancer, as well as of tongue cancer, pancreatic cancer, and in regenerating hepatocytes of newts [2]. Later, this phenomenon was reproduced by D. Brandes et al., who published in 1965 in *Science* a brief article entitled “Nuclear Mitochondria?” In their study, similar to that of Mori, cancer (leukemic) cells were used [3]. Since then, mitochondria in nuclei have been found in white blood cells [4,5], lymph nodes of patients with Hodgkin’s disease [6], leukemic myoblasts [7], in cardiomyocytes of a patient with rheumatic heart disease [8], and certain other cardiac pathologies [9,10,11,12]. Given that the presence of intranuclear mitochondria has been exclusively proven in abnormal cells, these facts were attributed to the manifestation of the pathology.

Two main issues elicit discussion: how do mitochondria get into the nucleus and what advantages or disadvantages arise as a result of such organelle interaction? Several explanations of such observations have been suggested. Most frequently, the appearance of mitochondria inside nucleus was assigned to the improper execution of mitosis. Using immunofluorescence techniques, it has been shown that a brief opening of the nuclear membrane can occur in the interphase nucleus. Nuclear membrane remodeling was found during viral infections [13,14,15], laminopathy [16,17,18], muscular dystrophy, cardiomyopathy, lipodystrophy [19,20], Hutchinson–Gilford progeria syndrome [21], and cancerogenesis [18,22,23,24,25]. However, it may be premature to consider this phenomenon as specific for pathological processes only. For example, mitochondria in nuclei were observed by Zhao et al. at the final stage of erythropoiesis in mice [26]. Immunofluorescence assays as well as focused-beam and scanning electron microscopy methods have shown that erythroblast nuclei can be in the opened and fragmented state for 3–5 min. The opening is followed by relatively stable periods of closure lasting about an hour with caspase-3 to be essential for this cyclic process. Loss of caspase-3 blocks not only the opening but also erythroid differentiation, leading to hematologic disorders.

There is no doubt that in terms of energy, nucleus function is quite costly in using, for many processes, cytosolic ATP, which is mostly generated by mitochondria. Limiting diffusion distance for intracellular ATP transport to the site of its use may be an issue to facilitate ATP transport directly to the site of priority use. On the other hand, mitochondria and the nucleus possess genomes of different nature and properties, and numerous data have reported on their interaction and cross-talk. A common opinion is that the transfer of mitochondrial DNA to the nucleus has contributed to the evolution of eukaryotic genomes [27,28,29]. Mitochondrial DNA transfer to the nucleus is an established fact, possibly playing both normal [30] and pathological [31,32] roles. Vice versa, anterograde signaling (from nucleus to mitochondria) includes numerous regulatory factors coordinating the function of subcellular organelles and integrating cellular and environmental signals, such as nuclear respiratory factor 1 (NRF1) [33], nuclear factor erythroid 2-like 2 [34] (NFE2L2 or NRF2), peroxisome proliferator-activated receptors (PPARs), and estrogen-related receptors (ERRs) [35], as well as many others that regulate mitochondrial-specific activities.

It is reasonable to consider that increasing nuclear membrane surface would facilitate the exchange rate between nucleoplasm and cytosol. Indeed, numerous deep and branching invaginations of the nuclear envelope [36,37], especially in cancer cells [38], were found.

Unlike nuclear envelope invaginations possibly serving as mechanism for importing cytosolic components to the nucleoplasm, envelope herniations may serve the opposite role through exporting nuclear content to cytosol [25,39]. Recently found mitochondria-derived vesicles [40,41] may play a role as a vehicle providing transport of genetic material to the nucleus.

In this study, we made an attempt to analyze the appearance of mitochondria in the nucleus, comparing the heart cells of two species of animals, radically different in life expectancy: rats and naked mole-rats (*Heterocephalus glaber*), as well as the line of rats named by the breeder as OXYS, characterized by accelerated aging. To analyze the structure of the mitochondrial network and its relationship with the nucleus, two microscopic techniques were used: confocal and electron microscopy. Confocal microscopy by itself is not able to resolve mitochondria in nuclear membrane invaginations of those residing in the nucleoplasm. A combination of confocal and electron microscopy may become not only the instrument to address this question but also it would help to address the functionality of nuclear mitochondria.

Indeed, our analysis using confocal microscopy revealed a specially organized functional mitochondrial network in the vicinity of the nuclei in normal cardiac myocytes, whereas electron microscopic images convincingly demonstrated the absence of nuclear membrane over relatively large areas of the nucleus. Regardless of the disruption of the nuclear membrane integrity, the content of the nucleus preserved its specific morphology. Here, we present the ultrastructure of open nuclei containing mitochondria in normal cardiomyocytes of Wistar and OXYS rats, as well as naked mole-rat (*Heterocephalus glaber*). The latter species is of particular interest because it is very long-lived and is resistant to many pathologies inherent in other species. Therefore, it can be an example of a mammal that has succeeded in maintaining a long and healthy life.

## 2. Results

**Figure 1:**
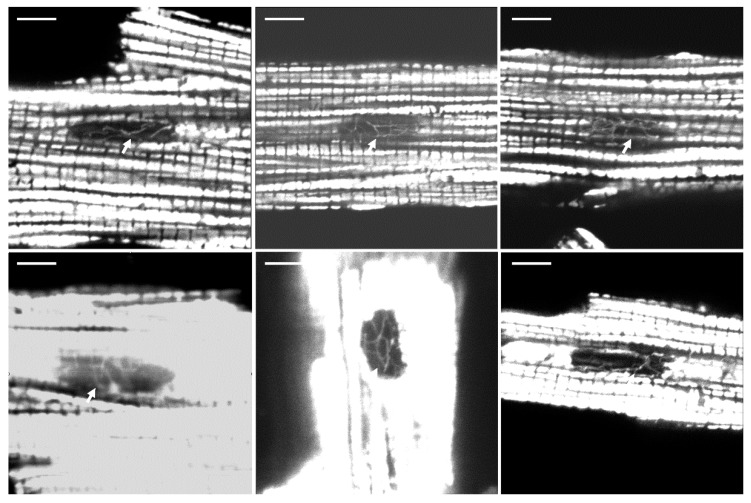
Variability of the mitochondrial architecture in the vicinity of nuclei in normal rat ventricular cardiac myocyte was stained with the mitochondria-targeted probe tetramethyl rhodamine methyl ester (TMRM; 200 nM). Confocal microscopy. Bright images represent energized mitochondria along myofilaments of the heart cell. We used a pinhole of 150 mµ allowing one to observe tilted mitochondrial chains spanning the cell, thus making an impression of the appearance and disappearance of these chains. In some images, in order to reveal the peri(intra)nuclear mitochondrial network (arrows), the detector gain was artificially enhanced, making the fluorescence of interfibrillar mitochondria saturated. Scale: 5 mµ.

**Figure 2:**
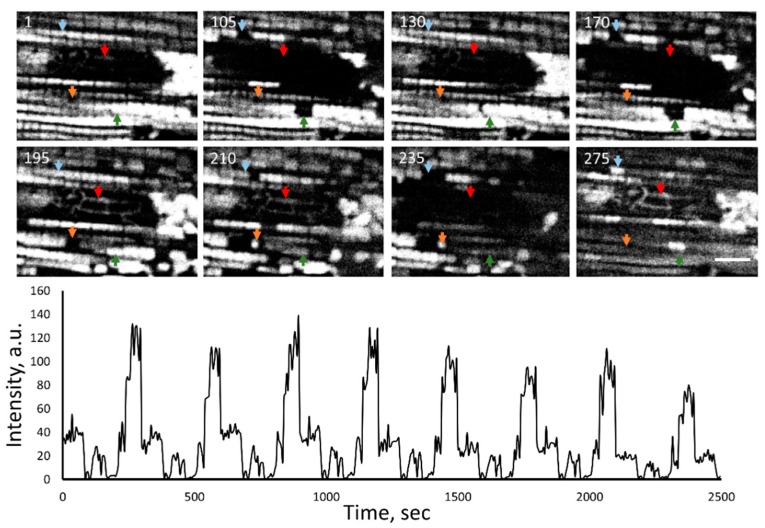
Photo-induced oscillations of the membrane potential in mitochondrial clusters within cardiac myocytes loaded with 200 nM TMRM. Colored arrows show some oscillatory elements at different time intervals indicated in the upper-left corner (in seconds) of each confocal scan. The example at the bottom shows periodic changes of the fluorescence intensity of TMRM in the region shown by the red arrow. Scale: 5 mµ. Full-time series of this sample is presented in Supplementary Video S2.

Confocal microscopy of a normal Wistar rat cardiac mitochondrial architecture in the vicinity of nuclei revealed a very complicated mitochondrial network organized by tiny branched mitochondrial filaments. Practically all cardiac nuclei were surrounded by a mitochondrial web, deeply penetrating the body of the nucleus (Figure 1, Supplementary Video S1). These visually observed structures were identified with a variety of mitochondrial dyes, at least one of these being potential-dependent tetramethyl rhodamine methyl ester (TMRM). Mitochondrial dye nonyl acridine orange (NAO), apparently interacting with mitochondrial cardiolipin independently of the existence of the membrane potential (Supplementary Video S4), as well as Mitotracker Deep Red (not shown), revealed the same peri(intra) nuclear mitochondrial network suggesting that these nuclear mitochondria are fully functional. To exclude that these structures belong to sarcoplasmic reticulum non-specifically stained with mitochondrial dyes, we used an approach of photo-induced oscillations of the mitochondrial membrane potential [42]. Observed oscillations of a different part of the mitochondrial reticulum including the peri(intra)nuclear part confirmed that these structures were mitochondria with maintained membrane potential (Figure 2, Supplementary Video S2). However, in spite of obvious very deep penetration of mitochondrial fluorescence images into the space occupied by the nucleus, the light microscopic level approach did not allow us to discriminate mitochondria deeply invaginated in the nucleus from those residing in the nucleoplasm. Subsequent electron microscopic study of the normal cardiac myocyte was designed to resolve this question.

**Figure 3:**
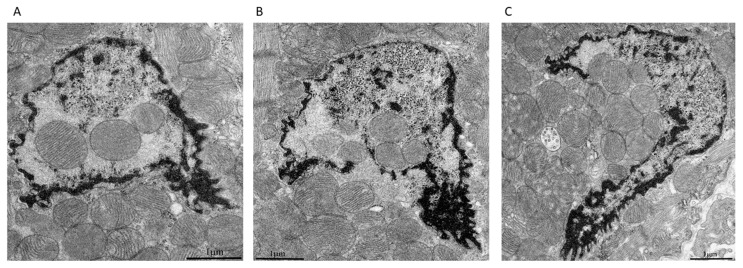
(**A**–**C**) Consecutive ultrathin sections from serial sections of a cardiomyocyte nucleus of a 3-month-old Wistar rat. Electron microscopy.

**Figure 4:**
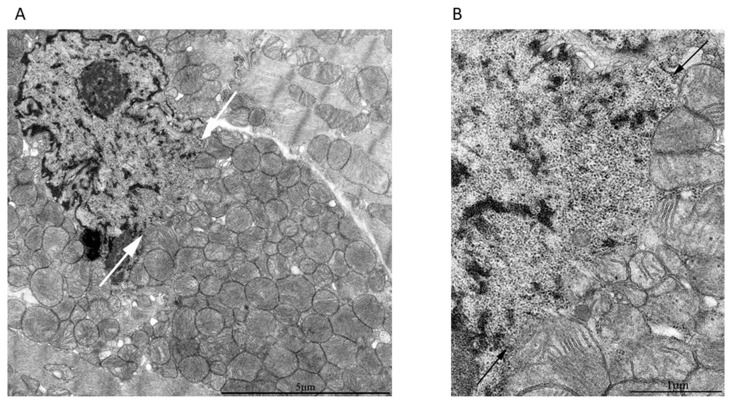
(**A**) Direct contact of a mitochondrial cluster with nuclear structures in a cardiomyocyte from a 24-month-old OXYS rat. The area of contact is indicated by an arrow. (**B**) The local fragment of the nucleus indicated by the arrows in Figure 4A. The nuclear membrane was absent, and individual mitochondria were directly adjacent to the nucleoplasm.

**Figure 5:**
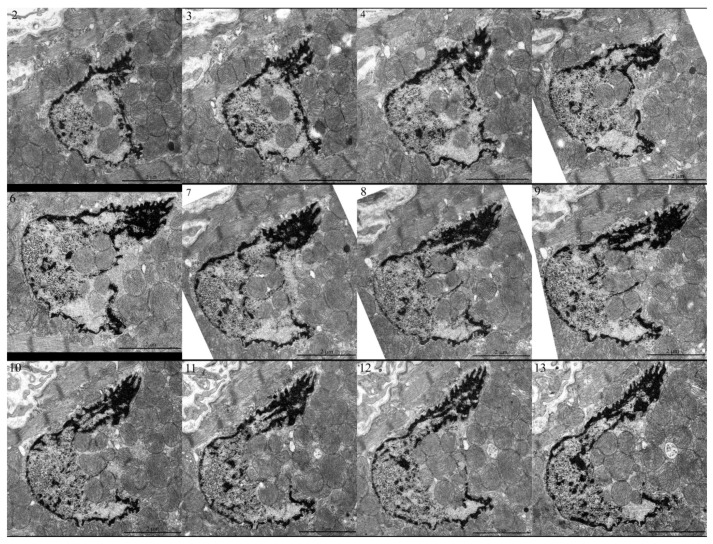
Serial micrographs of 12 sections over the nucleus of the cardiomyocyte of a 3-month-old Wistar rat with mitochondria embedded in the nucleoplasm.

Figure 3A–C represents three consecutive ultrathin sections of cardiac myocytes of a 3-month-old Wistar rat. As shown in Figure 3A, three mitochondria were clearly visible inside the nucleus. The mitochondria were not separated from the contents of the nucleus by the nuclear membrane, that is, they, in fact, were located in the nucleoplasm. In the subsequent sections of this nucleus, the number of mitochondria inside the nucleus was increased. In Figure 3B,C, the contents of the nucleus were in direct contact with a mitochondrial cluster due to the partial absence of the nuclear membrane. It should be noted that in Figure 3A,B, the nuclear area directly surrounding the mitochondria had a fine fibrillar structure differing greatly from the granular structure in the main part of the nucleoplasm. In this case, a fragment of cytoplasm containing mitochondria was supposed to enter the nucleus through the open aperture in the nuclear membrane. Figure 4A,B show direct contact between a mitochondrial cluster and nuclear structures in a cardiomyocyte of a 24-month-old OXYS rat. Furthermore, using heart samples from a 3-month-old Wistar rat, we performed a three-dimensional reconstruction of a part of the nucleus with the mitochondria present inside, showing the architecture of the chromatin and nuclear membrane on the basis of the analysis of a sequential series of ultrathin sections for electron microscopy (Figure 5 and Supplementary Movie 3).

**Figure 6:**
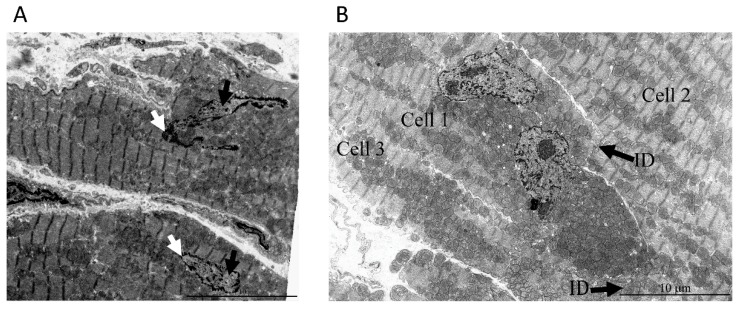
Ultrastructures of nuclei and cytosols of the rat heart tissue with cells, one of which contained intranuclear mitochondria while the others did not. (**A**) Ultrastructure of cardiomyocytes from a 3-month-old Wistar rat with mitochondria in the nucleus (upper cell) and without them (lower cell). White arrows indicate condensed chromatin and black arrows indicate decondensed chromatin. (**B**) Cytosolic ultrastructure of the 24-month-old OXYS rat cell with intra-nuclear mitochondria (cell 1) and adjacent cells (cell 2 and cell 3), connected by intercalated discs (ID). Note that (**B**) is a low zoom of the cardiac tissue containing the region depicted in Figure 4A,B in the manuscript.

The main and very important argument is that this particular examined cell with the intranuclear mitochondria was abnormal. However, this was not confirmed by the ultramicroscopic characteristics of the nucleus and the cytoplasm surrounding this nucleus. We compared the ultrastructure of these cells containing the obvious intranuclear mitochondria with ultrastructure of cells where intranuclear mitochondria were missing and found no significant alterations indicating cell damage (Figure 6).

In the nuclei of cardiomyocytes with nuclear ruptures, chromatin was preferentially decondensed, and the condensed chromatin was visible at nuclear periphery in close contact with the nuclear envelope, around the nucleoli (perinucleolar chromatin), and inside the nucleoplasm. The blocks of condensed chromatin in the nucleoplasm adjusting the nuclear envelope ruptures had elongated shape, probably due to mechanical tension. The similar localization of condensed chromatin was detected in the nuclei without nuclear envelope ruptures. This apparently mechanical deformation of chromatin complexes was visible near nuclear envelope ruptures, indicating that nuclei were under a strong pulling force action, which potentially could induce these ruptures. Decondensed chromatin was not substantially modified, even in regions that were in direct contact with the mitochondria. Thus, in the nuclear regions adjacent to broken nuclear membrane, the changes in chromatin were minimal, whereas on the nuclear periphery far from these regions, the chromatin configuration was not distinguished in both types of cells (Figure 6A).

As for the cytosolic ultrastructure of the cell with intranuclear mitochondria (Figure 6B), the ultrastructure of myofibrils was conventional with regular position of isotropic and anisotropic regions separated by a Z-line. Myofibrils are longitudinally oriented and densely packed. Sarcomeres have a conventional size of 2–3 microns in length. Intercalated disks are not damaged with a typical structure. The sarcoplasma is not swollen with mitochondria having a normal orthodox structure.

**Figure 7:**
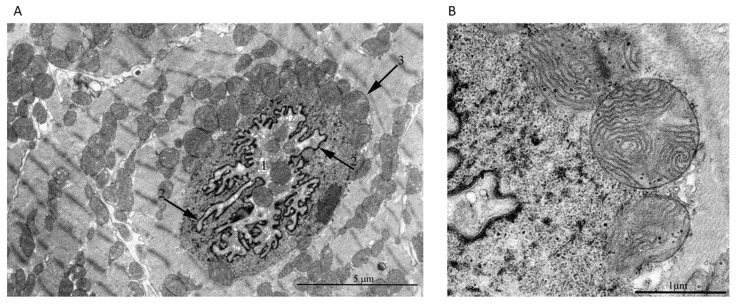
(**A**) Direct contact of mitochondria with nuclear structures in a cardiomyocyte from a 5-year-old naked mole-rat. In this section, the nuclear membrane was absent along the entire perimeter of the nucleus. Arrow 1 shows the cytoplasm with organelles including mitochondria was located inside the nuclear invagination. Arrow 2 shows the nuclear membrane of the nucleus invagination. (**B**) A group of mitochondria shown by arrow 3 in Figure 7A at high magnification. Mitochondria were in direct contact with nuclear structures and were submerged in the nucleoplasm.

**Figure 8:**
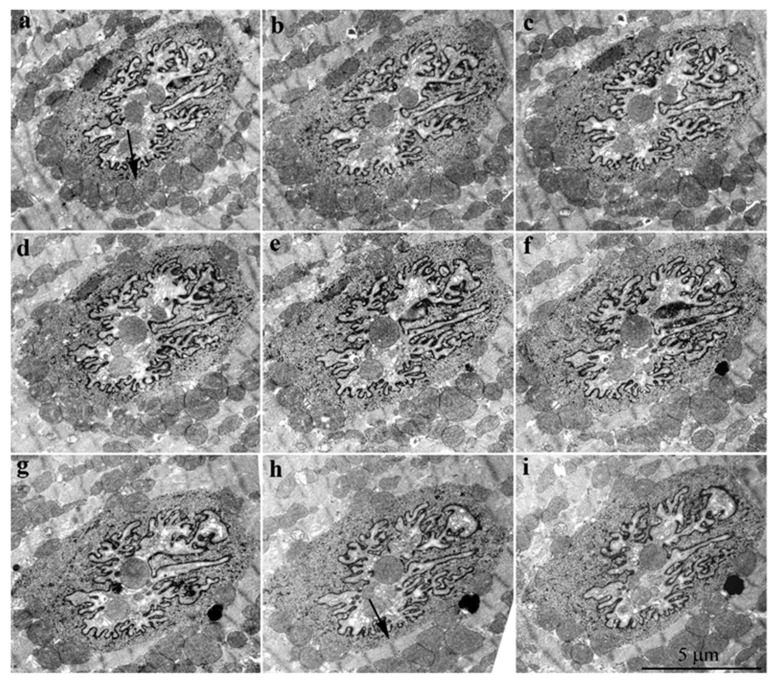
Serial of consecutive sections of the nucleus of a cardiomyocyte (from **a** to **i** with the thickness of each section ~500 Å) from the 5-year-old naked mole-rat presented in Figure 7A. Arrows in sections **a** and **h** point to the same area of the nucleus, proving that mitochondria did not form a continuous layer contacting the nuclear contents. In section **a**, the mitochondria were in direct contact with nuclear structures, whereas in section **h**, there was direct contact of nuclear content with adjacent myofibrils.

A more striking picture of a direct contact between mitochondria and structures of the nucleus was found in a cardiomyocyte of a 5-year-old naked mole rat (Figure 7A,B). In this case, the sections were made in such a way that the absence of the nuclear membrane was revealed along the entire perimeter of the nucleus. The specific morphology of the nucleus was preserved despite the vast area lacking the nuclear membrane. Figure 7B shows at higher magnification the mitochondrial group indicated by arrows in Figure 7A. It is clearly seen that the mitochondria were in direct contact with the intranuclear structures. The analysis of serial sections of this nucleus (Figure 8) revealed that mitochondria did not form a continuous layer contacting the contents of the nucleus. There were some nuclear areas directly adjacent to myofibrils (indicated by the arrow, Figure 8, section h).

The statistical analysis of the occurrence of mitochondria in cardiomyocyte nuclei showed that, on average, the percentage of nuclei with mitochondria was roughly around 1%, and it did not show age and species dependency (see Supplementary Table S1).

## 3. Discussion

The presence of mitochondria in the nuclei was claimed more than 50 years ago [1,2,3], but the objects used for these studies belonged to pathological tissues. This was the reason to assign such a feature to a pathological symptom. In addition, these data were criticized due to the poor quality of the sample and the possibility of artifacts caused by improper treatment of the sample as part of the electron microscopic technique. Penetration of mitochondria into the nucleus as a result of mechanical tissue damage occurring during fixation was discussed by Takemura et al. [10], who found mitochondria in nuclei of myocardial cells taken from patients with various cardiac diseases. However, mitochondria were found in the nucleus of cultured cells, where, due to specific fixation techniques, mechanical damage did not occur.

A reasonable explanation of the presence of mitochondria inside a nucleus was improper execution of mitosis. However, this explanation was not suitable for cardiac myocytes, which belong to postmitotic cells. Several other observations have recently been made that disprove the assertion that invasion of mitochondria to the nucleus occurs when the nuclear membrane is disassembled during mitosis [23,24,25,26].

Confocal microscopy revealed mitochondrial organization in the vicinity of a nucleus, which was different from the well-known interfibrillar and subsarcolemmal mitochondrial population. Mitochondrial web consisting of thin branched filaments covering all peri(intra)nuclear space was typical for all explored normal nuclei of isolated rat ventricular cardiac myocytes. 3D reconstruction of the space occupied by a nucleus demonstrated deep sprouting of mitochondrial filaments into this space (see Supplementary Movie 1). All mitochondrial filaments were fully functional because they were stained with membrane potential sensitive dye and specific mitochondrial marker cardiolipin, and responded to photoexcitation by the partially reversible oscillations of the mitochondrial membrane potential (see Supplementary Movie 2).

Using electron microscopy, we concluded that there was direct contact between mitochondrial clusters and nucleoplasm in cardiomyocytes of the healthy rodents: 3-month-old Wistar rat, 24-month-old OXYS rat, and 5-year-old naked mole rat. Serial ultrathin sections of the same nucleus showed that, depending on the section level, it was possible to observe mitochondria either inside of the closed nucleus or inside of the open nucleus partially devoid of the nuclear membrane. Statistics showed that 1–2% of nuclei present on ultrathin sections of cardiomyocytes contained mitochondria. Our findings are in line with the findings of a Norwegian research group who reported mitochondria in 2–3% of cardiomyocyte nuclei in a patient with rheumatic heart disease [8]. The observations, first made already in the middle of the 20th century of mitochondria inside a nucleus, are no longer considered an artifact of electron microscopy technique [42]. On the basis of a great number of immunofluorescence assays in which brief disruption of the nuclear membrane in interphase nuclei was observed in association with various diseases and abnormal conditions [18,22,23,24,25] as well as in healthy cells [26,43], the presence of mitochondria in the nucleoplasm is usually considered as a result of catastrophic loss of the barrier function of the nuclear membrane that might be a contributing factor of disease progression [44]. In some reports, the penetration of mitochondria into the nucleus was believed to occur due only to a mechanical process [12,44]. It was suggested that the constant contractile function in cardiomyocytes contributes to the penetration of mitochondria into the nucleus through a pathology-weakened nuclear membrane. However, in this study, we showed that mitochondria appeared in the nucleus of normal cardiomyocytes. On the basis of the 3D reconstruction of a part of the cardiomyocyte with nuclei-containing mitochondria, we conclude that the nuclear membrane could be absent in the extensive nuclear region and that it is represented by patches. In all our cases, we describe the presence of mitochondria in nuclei having open configuration without nuclear membrane resealed.

We were unable to answer the question of how specific this discovered phenomenon is for cardiomyocytes. There is evidence that the nuclear membrane undergoes structural changes during mechanical action, which are expressed in local loss of the nuclear envelope integrity [45,46]. This was especially well-observed in the example of migration of cancer cells through narrow holes that led to deformation of the nuclei combined with local breaks of the nuclear membrane [25], which allowed simulating the situation by direct physical actions on the cell [47,48]. Thus, chronic mechanical effects on the cell nucleus [49], associated with contractile activity of the heart, could be the cause of similar changes in the structure of the nucleus, leading to local damage/elimination of the nuclear envelope. Cardiomyocytes are cells chronically exposed to a deforming challenge, with mitochondria changing their shape under a normal cardiomyocyte twitch, caused by the dynamic force-balance inside cardiomyocytes and by changes in the spatial stiffness characteristics [50]. A similar mechanotransduction at the nuclear level was observed in endothelial cells a priori exposed to a chronic shear stress [51].

In 2016, Zhao et al. were the first to study the functional significance of nuclear membrane remodeling in interphase nuclei during erythropoiesis in mice [26]. They showed that this process is not accompanied by a dramatic release of nuclear components into the cytoplasm leading to the loss of cell functions and cell viability, as previously supposed [44]. They showed that the dynamic nature and cyclic repetition of nuclear opening are essential for normal differentiation, ensuring the release of nuclear histones into the cytoplasm for chromatin to be condensed during terminal erythropoiesis. They showed that the release of histones into the cytoplasm is a selective process, and that non-histone nuclear proteins stay inside the nucleus. The released nuclear histones accumulate around the open fragment of the nucleus, performing a protective function, blocking the release of non-histone nuclear proteins, and supporting nuclear/cytoplasmic compartmentalization.

We suppose that local nuclear membrane disassembling, which we observed in cardiomyocytes, as well as subsequent contact between mitochondria and nuclear contents, are of functional significance. Unfortunately, at present, it is impossible to trace the fate of such nuclei and cardiomyocytes containing them. However, during terminal erythropoiesis, Zhao et al. [26] proposed the necessity of the nuclear opening process. As follows from the ultrastructural picture of open nuclei in erythroblasts presented by those authors, the contact between nuclear and cytoplasm components along relatively large areas of the nucleus lacking the nuclear membrane does not lead to cell pathology or apoptosis. In this connection, it is important to mention reports in which authors discovered the direct contact of mitochondria with nuclear components in *Ciona internalis* oocytes [52], as well as with nucleus-like bodies in *Rana pipiens* oocytes [53], and authors have even described special filaments mediating the association of mitochondria with nuclear structures.

It seems that so-called open nuclei, as well as the presence of mitochondria inside nuclei, are a natural and common biological phenomenon related to mitochondrial/nuclear interactions. In eukaryotic cells, mitochondria take part in intracellular regulations mediated by cross-talk between mitochondria and the nucleus. This interaction is represented by anterograde (nucleus–mitochondrion) and retrograde (mitochondrion–nucleus) signaling [32]. This cross-talk includes exchange by ATP/ADP, regulatory proteins and genetic material going in both directions. Bidirectional transport of genetic material is of primary interest for molecular biologists due to its high relevance to the evolution of eukaryotic genomes [27,28,29] and the occurrence of diseases through regulation of gene expression, possibly by non-coding RNAs originating both from mitochondria [32] and nuclei [31,32,33,34,54,55,56,57]. The so-called “escape” of nucleic acids from nuclei and mitochondria [58] seems to be part of a well-designed strategy of communication of genomes rather than being an occasional stochastic process. Shortening the distance between genomes will not only facilitate their interaction but also reduce the probability of degradation; in particular, cytosolic nucleases and penetration of mitochondria in the nucleus might serve this strategy.

## 4. Materials and Methods

Animals: 3- and 24-month-old male senescence-accelerated OXYS and Wistar rats were obtained from the Shared Center for Genetic Resources of the Institute of Cytology and Genetics (ICG), Siberian Branch of the Russian Academy of Sciences (Novosibirsk, Russia). The OXYS rat strain was established on the basis of the Wistar rat strain at the Institute of Cytology and Genetics, as described earlier [59], and registered in the Rat Genome Database (http://rgd.mcw.edu/). At the age of 4 weeks, the pups were taken from their mothers, housed in groups of five animals per cage (57 × 36 × 20 cm), and kept under standard laboratory conditions (at 22 ± 2 °C, 60% relative humidity, and natural light), provided with standard rodent food, PK-120-1, Ltd. (Laboratorsnab, Russia). Naked mole rat colonies were maintained at the Leibniz Institute for Zoo and Wildlife Research, Berlin, Germany, in an artificial burrow system with plexiglass tunnels and boxes. The system was heated to 26–29 °C with constant humidity of 60–80%. The chambers contained wood bedding, twigs, and unbleached paper tissue. Fresh food was given daily ad libitum. It included sweet potatoes, carrots, fennel, apples, a cereal supplement containing vitamins and minerals, and oat flakes. The local ethics committee of the “Landesamt für Gesundheit und Soziales”, Berlin, Germany (#ZH 156), approved sampling.

### 4.1. Cardiac Myocytes Isolation

Ventricular cardiac myocytes used in the study were isolated from adult Wistar rats (2–4 mo old) by a standard enzymatic technique [60] through initial perfusion of hanged isolated heart with the medium containing collagenase type II and subsequent breakage of digested heart pieces by a gentle pipetting and transfer to media with growing Ca^2+^ content. Final HEPES-buffered solution contained (in millimoles per liter) 137 NaCl, 4.9 KCl, 1.2 MgSO_4_, 1.2 NaH_2_PO_4_, 15 glucose, 20 HEPES, and 1.0 CaCl_2_, pH 7.4.

### 4.2. Confocal Microscopy

Myocytes were loaded with dyes for >20 min on a glass-bottom Petri dishes, incubated in HEPES-buffered solution (same composition as the storage solution) at 23 °C, and imaged with a LSM-510 inverted confocal microscope using a Plan-Neofluar 63 ×/1.25N.A. oil immersion lens (Carl Zeiss Inc., Jena, Germany). Scans were recorded in a single channel mode with excitation at 543 nm (for tetramethyl rhodamine methyl ester (TMRM; Molecular Probes, Inc., Eugene, OR, USA), nonyl acridine orange (NAO; Sigma-Aldrich, St. Louis, MI, USA), and mitotracker Deep Red (MTDR; ThermoFisher Scientific, Waldham, MA, USA)), collecting simultaneous fluorescence emission with LP560, LP 505, and LP650 nm, respectively. The confocal pinhole was set to obtain spatial resolutions of 0.4 μm in the horizontal plane and 1.0 μm in the axial dimension. Image processing was performed using Fiji software (U.S. National Institutes of Health, Bethesda, MD, USA). Frame scan along z-direction was performed to cover the entire space occupied with nuclei, which was identified as space of a spheroid shape (usually two per cell) poor in mitochondria. Time series mode along a single *x*–*y* plane was performed with 5 second intervals between scans.

### 4.3. Electron Microscopy

For electron microscopic investigation, samples were fixed with 3% glutaraldehyde solution (pH 7.4) for 2 h at 4 °C, over-fixed with 1% osmium tetraoxide solution for 1.5 h, and then dehydrated in alcohol series with increasing alcohol concentrations (70% alcohol was saturated with uranyl acetate). The samples were embedded in Epon-812 epoxy resin. Serial ultrathin sections were made with a Leica ULTRACUT UCT microtome and stained by lead according to Reynolds [61]. The resulting preparations were scanned and photographed using a JEM-1400 electron microscope (JEOL, Japan).

## Data Availability

Typical results of this study are included in this published article.

## Supplementary Materials

The following are available online at https://www.mdpi.com/2073-4409/9/3/712/s1: Video S1. 3D reconstruction of the mitochondrial network from rat ventricular cardiac myocyte stained with the tetramethyl rhodamine methyl ester (TMRM). Video S2. Timeseries of the mitochondrial network from rat ventricular cardiac myocyte stained with tetramethyl rhodamine methyl ester (TMRM). Video S3. 3D reconstruction of the mitochondria from ultrathin sections from serial sections of a cardiomyocyte nucleus of a 3-month-old Wistar rat. Video S4. 3D reconstruction of the mitochondrial network from rat ventricular cardiac myocyte stained TMRM. Table S1. The frequency of mitochondrial appearance in nuclei of cardiomyocytes of Wistar, Oxys and naked mole rats of different ages.
