# Supplementary material for: Mitochondria in the Nuclei of Rat Myocardial Cells
Source: Cells. 2020 Mar 14;9(3):712. doi: 10.3390/cells9030712 (PMC7140638; doi:10.3390/cells9030712)
Supplement: Supplementary file 1 [file cells-09-00712-s001.zip › sup_skulach/Table 1.docx]

Table 1. The frequency of mitochondrial appearance in nuclei of cardiomyocytes of Wistar, Oxys and naked mole rats of different ages.

|  | Wistar rats | | OXYS rats | | Naked mole rat |
| --- | --- | --- | --- | --- | --- |
| Age | 3 m. o. | 24 m. o. | 3 m. o. | 24 m. o. | 6 m.o. |
| Total nuclei | 3099 | 3002 | 2912 | 2976 | 3119 |
| Nuclei w/mitochondria | 36 | 38 | 29 | 39 | 26 |
| St.error | 1,161665 | 1,265823 | 0,995879 | 1,310484 | 0,833600513 |
|  |  |  |  |  |  |
