# Supplementary figures and images for: Mitochondria in the Nuclei of Rat Myocardial Cells
Source: Cells. 2020 Mar 14;9(3):712. doi: 10.3390/cells9030712 (PMC7140638; doi:10.3390/cells9030712)

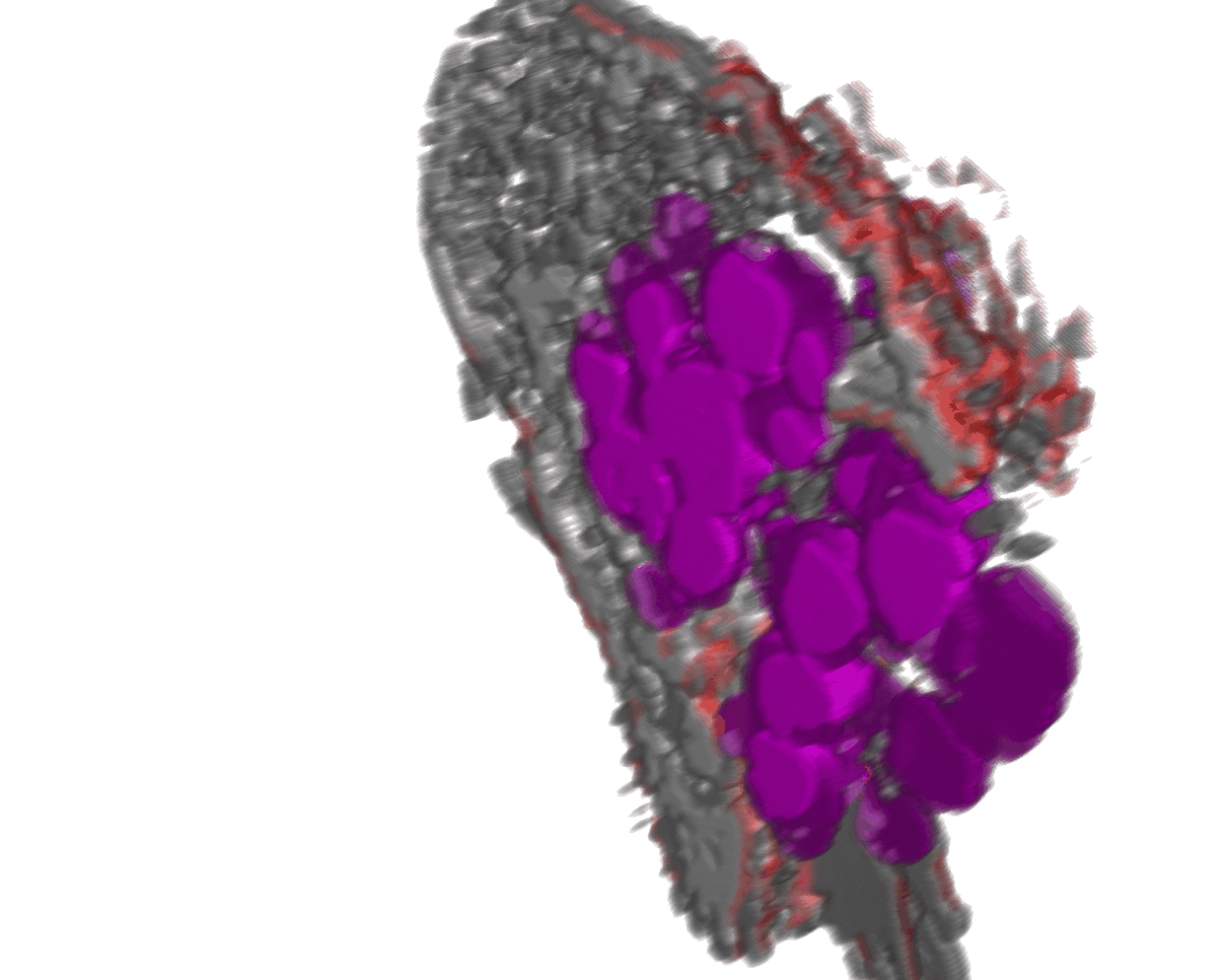

Supplement: Supplementary file 1 [file cells-09-00712-s001.zip › sup_skulach/video 3.gif]

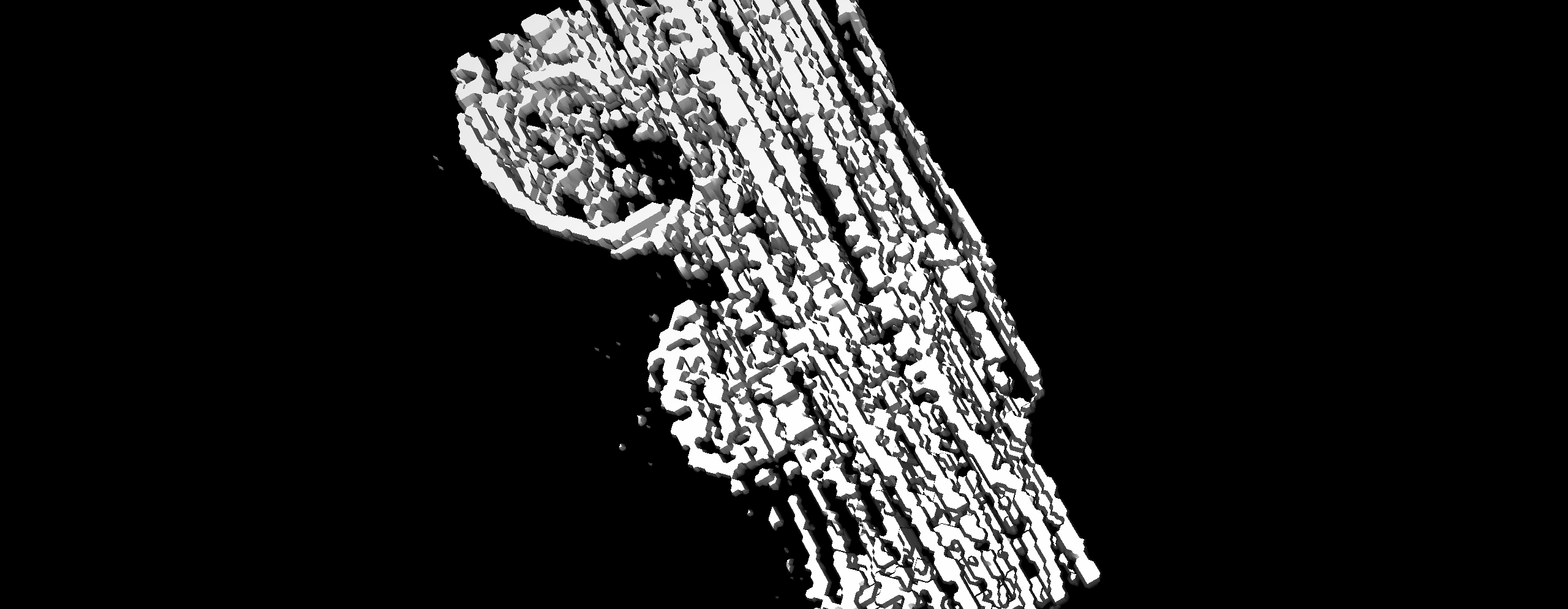

Supplement: Supplementary file 1 [file cells-09-00712-s001.zip › sup_skulach/video 4.gif]

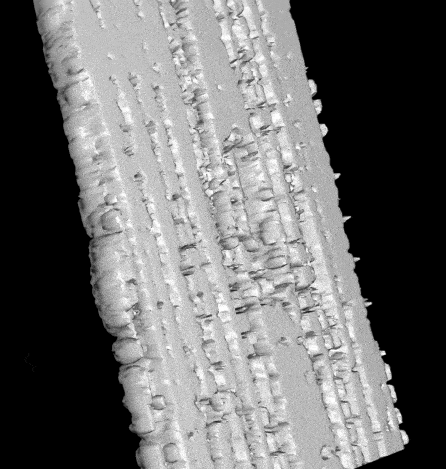

Supplement: Supplementary file 1 [file cells-09-00712-s001.zip › sup_skulach/video1.gif]

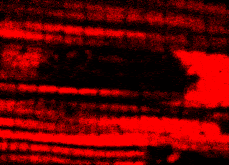

Supplement: Supplementary file 1 [file cells-09-00712-s001.zip › sup_skulach/video2.gif]
